# Previous Interspecific Courtship Impairs Female Receptivity to Conspecifics in the Parasitoid Wasp *Nasonia longicornis* But Not in *N. vitripennis*

**DOI:** 10.3390/insects9030112

**Published:** 2018-09-01

**Authors:** Magdalena M. Mair, Nicole Seifert, Joachim Ruther

**Affiliations:** Institute of Zoology, University of Regensburg, Universitätsstraße 31, D-93053 Regensburg, Germany; magdalena.mair@ur.de (M.M.M.); nicole.seifert@stud.uni-regensburg.de (N.S.)

**Keywords:** behavioural plasticity, mate discrimination, parasitic wasp, reproductive interference, sympatry

## Abstract

Interspecific sexual interactions are not uncommon in animals. In sympatry, females often face the risk of accidentally mating with a heterospecific male. Based on the actual risks imposed by the environment at a given time and place, females should be able to adjust their mate acceptance in order to avoid interspecific copulations as well as accidentally refusing to mate with a conspecific. We investigate the ability of females of the two parasitoid wasp species *Nasonia vitripennis* (*Nv*) and *N. longicornis* (*Nl*) to adjust their mate acceptance in response to previous unsuccessful courtship by heterospecific males. We show that *Nl* females are more reluctant to mate with a conspecific male when having been courted previously by a heterospecific male, but *Nv* females are not. We argue that this strategy is reasonable for *Nl* females but not for *Nv* females, which follow a different strategy to avoid the fitness costs imposed by heterospecific copulations.

## 1. Introduction

Reproductive interference is common among a wide variety of animal taxa [1]. Due to incomplete species recognition during mate acquisition, signals sometimes attract individuals of the wrong species [2,3], courtship is directed towards the wrong mating partners [4], males try to copulate with heterospecific females [5], and females occasionally become receptive to heterospecific males [6]. To avoid the fitness costs arising from interspecific copulations, the ability to discriminate between conspecific and heterospecific courtship partners usually evolves in the choosing sex (usually females) [7]. During mate acquisition, a female assesses the species of the courting male, and refrains from copulation if the male does not belong to the same species. Mate discrimination acts as an important prezygotic hybridisation barrier [8,9], and is particularly important in species in which females mate only once during their lifetime and post-mating reproductive isolation is complete [10]. In insects, mate discrimination usually involves chemical messengers [11], but it frequently includes additional signals, e.g., specific courtship displays, acoustic signals, or wing vibration patterns [12]. Closely related species often resemble each other in their courtship display, and mate discrimination is not absolutely accurate [13,14,15]. Females face both the risk of accidentally mating a heterospecific male and the risk of accidentally rejecting a conspecific. A trade-off thus arises between either becoming more selective in order to prevent interspecific copulations or broaden the range of stimuli that elicit receptivity in order to avoid accidentally rejecting a conspecific. Females should minimise both of these risks by adjusting their behaviour depending on the actual risks and costs imposed by the environment at a given time and place. If the chance of meeting and being courted by a heterospecific partner is low, e.g., in areas of allopatry, the costs of rejecting a conspecific become comparably large, and the range of partners accepted for copulation should be broadened. In contrast, in sympatry, the chance of being courted by a heterospecific male increases with the increasing population density of the interfering species, and interspecific copulations become more likely. In these situations, it is advantageous for females to become more selective by either establishing more accurate discrimination abilities or accepting the accidental rejection of a conspecific in order to avoid the much higher costs of consenting to interspecific copulation. It has been shown in various animal taxa that when animals are reared in sympatry with an interfering species, mate discrimination abilities can become more accurate through learning by experience either early in their life (e.g., sexual imprinting) or at later stages of their lives (e.g., contextual behavioural plasticity) [16,17,18]. One means by which females may learn about the risks of interspecific mating in a specific environment is the experience of being courted by a heterospecific male. However, nothing is known to date about the direct effects of heterospecific courtship experience on future female mate acceptance.

*Nasonia vitripennis* (*Nv*) and *N. longicornis* (*Nl*) are two species of parasitoid wasps that parasitise the pupae of cyclorrhaphous flies. In the western part of North America, *Nv* and *Nl* occur frequently in microsympatry, i.e., they develop within the same host individual [19,20,21,22]. As fly pupae often occur in a clumped distribution, and *Nasonia* wasps are gregarious, i.e., they lay more than one egg per host individual, males and females of *Nv* and *Nl* likely encounter after emergence at the same host patch. Courtship and copulation in *Nasonia* happen typically at the natal host patch, and females leave after mating to search for new oviposition sites [23,24,25]. In laboratory studies, males readily engage in courtship with females of the other species [25,26,27,28]. Males exhibit an elaborate courtship display, including specific movements of their forelegs over the female’s head accompanied by series of head-nodding movements during which a sex pheromone is transferred from the male’s oral glands to the female’s antennae [29,30]. The female shows receptivity by lowering her antennae and opening the genital orifice, and copulation follows. Although following the same general pattern, male courtship displays differ in detail between the different *Nasonia* species [29,31,32,33]. Females are able to discriminate between conspecific and heterospecific mating partners, but mistakes in mate discrimination occur [25,26,27,28,34]. In behavioural bioassays, *Nv* females usually discriminate more strongly against *Nl* males (more than 70% rejection of heterospecific males) than vice versa (less than 40% rejection of heterospecific males), and mate discrimination differs among different *Nasonia* strains [26,27]. However, differences in mate discrimination among *Nv* strains seem to be independent from the origin of these strains from areas of sympatry or allopatry with other *Nasonia* species [27]. *Nv* and *Nl* show complete postzygotic reproductive isolation due to *Wolbachia-*mediated cytoplasmic incompatibility, preventing the production of hybrid females [35]. Similar to most Hymenoptera, *Nasonia* are haplodiploid, and eggs fertilised by heterospecific sperm either die or develop into male offspring, similar to unfertilised eggs [35,36]. In addition, *Nasonia* females mate only once during their lifetime in nature [24]. Females having copulated with a conspecific male usually refrain from mating again [37]. Females consenting to interspecific copulation thus face particularly high fitness costs [10]. *Nv* females counteract these costs by increased remating with a conspecific male after having copulated with a heterospecific [25]. However, this effect has not been shown for *Nl*. Considering the fitness costs imposed on the females of the less discriminating species, *Nl*, it would be advantageous for them to adjust their mate acceptance behaviour depending on the actual presence or absence of heterospecific males.

Here, we investigate the impact of previous unsuccessful heterospecific courtship on the females’ acceptance of conspecific mates in *Nv* and *Nl*. We hypothesize that females of *Nl* use the experience of heterospecific courtship to adjust their mate acceptance behaviour. In particular, we hypothesize that *Nl* females that have been unsuccessfully courted by a heterospecific male are subsequently more reluctant to mate in general in order to avoid accidentally copulating with the wrong male. We expect this reluctance to be reflected in both a decrease in conspecific mating rate and an increase of the duration of courtship necessary to induce receptivity in couples where copulation happens. For *Nv* females, we hypothesize that if they adjust their mating rates, they do so to a lesser degree because, firstly, *Nv* females show stronger mate discrimination in general, and secondly, they counteract costs of heterospecific matings by increased remating with a conspecific. We address these hypotheses by performing mating trials with *Nv* and *Nl* females without prior contact to any male, and with females that have been courted previously by a heterospecific male.

We found that *Nl* females, but not those of *Nv*, decreased conspecific mate acceptance after having been courted by a heterospecific male. We argue that this strategy is advantageous for *Nl* females to avoid future mismating and that the difference in behavioural plasticity between the two *Nasonia* species is reasonable, considering that *Nv* females, as shown in earlier studies, follow a different behavioural strategy to counteract the costs imposed by interspecific copulation through increased remating.

## 2. Materials and Methods

### 2.1. Strains, Rearing, and Preparation of Wasps

Experiments were performed with the *Nv* strain NvHVRx [38] and the *Nl* strain NLMN8510*. Wasps were reared on freeze-killed pupae of the green bottle fly *Lucilia caesar* at 25 °C under a 16:8 light:dark regime. For behavioural bioassays, wasps were isolated from host puparia at their pupal stage, separated, and kept singly in 1.5-mL microcentrifuge tubes until being used in experiments. By isolating wasps at this developmental stage, it was ensured that adult wasps were unmated and had not had any direct contact with other adult wasps when the experiments began. Each day in the morning, isolated wasps were checked for emergences. Emerged wasps were sexed and defined as zero days old. For bioassays, zero-day-old females and one to three-day-old males were used.

### 2.2. Behavioural Bioassays

Mating trials were conducted in a standard mating arena consisting of a round hole (10 mm diameter, 3 mm height) cut into an acrylic glass plate and covered by a cover slip (for a detailed description of the arena, see Ruther et al. in 2000 [39]). In each mating trial, a female and subsequently a male were put into the arena. The arena was closed with a cover slip, and the couple was observed for five minutes. The females of each species were subjected to one of two treatments: (1) Females were tested with conspecific males without prior contact to any other individual. (2) Females were exposed to a heterospecific male for five minutes, and were eventually courted by the male. If the female did not show receptivity to the heterospecific male, the heterospecific male was removed, and the female was subsequently exposed in a second mating trial to a conspecific male. For each conspecific couple, it was noted whether the female consented to mating or not. If copulation happened, the duration of preceding courtship, i.e., the time span between the male mounting the female and the female’s receptivity signal (opening the genital orifice), was recorded.

Trials in which males (conspecific or heterospecific) did not engage in courtship were discarded. Trials in which the female showed receptivity to the heterospecific male were excluded from further treatment, but were noted in order to assess whether the exclusion of these females led to a bias in the experimental females. Excluding females that consented to interspecific mating could have potentially resulted in testing only those females in treatment two that were in general more reluctant to mate. However, only three *Nv* females (3.6%) and four females of *Nl* (4.8%) copulated with heterospecific males. Thus, it is unlikely that excluding these females resulted in a significant bias among the experimental groups.

Some *Nv* males exhibited aggressive behaviours towards *Nl* females. For each trial with *Nl* females, it was therefore noted whether the female was treated aggressively or not. Females were defined as having been treated aggressively when males, after having started courtship, turned their wings into a vertical position, jumped towards the female, and repeatedly grabbed the female, occasionally involving injuries, i.e., tearing off parts of the female’s legs or antennae. In these occasions, females usually crouched down and tried to run away from the respective males.

Each individual was tested only once. The assignment of individuals to treatments was randomised, and the order of treatments followed a blocked design. In total, 80 replicates were performed for each of the four treatments (*Nv* courted previously, *Nv* without prior contact, *Nl* courted previously, and *Nl* without prior contact).

### 2.3. Statistical Analysis

Conspecific mate rejection rates were compared between females having been courted previously and those without prior contact to heterospecific males using a 2 × 2 Chi-square test for each of the species separately. Differences in the duration of courtship were compared between females having been courted previously and those without prior contact to heterospecific males with a Mann–Whitney U test. In addition, conspecific mate rejection rates and the duration of courtship were compared between *Nl* females that had been subjected to aggressive versus non-aggressive heterospecific contact with a 2 × 2 Chi-square test and a Mann–Whitney U test, respectively.

## 3. Results

### 3.1. Conspecific Mate Rejection

**Figure 1:**
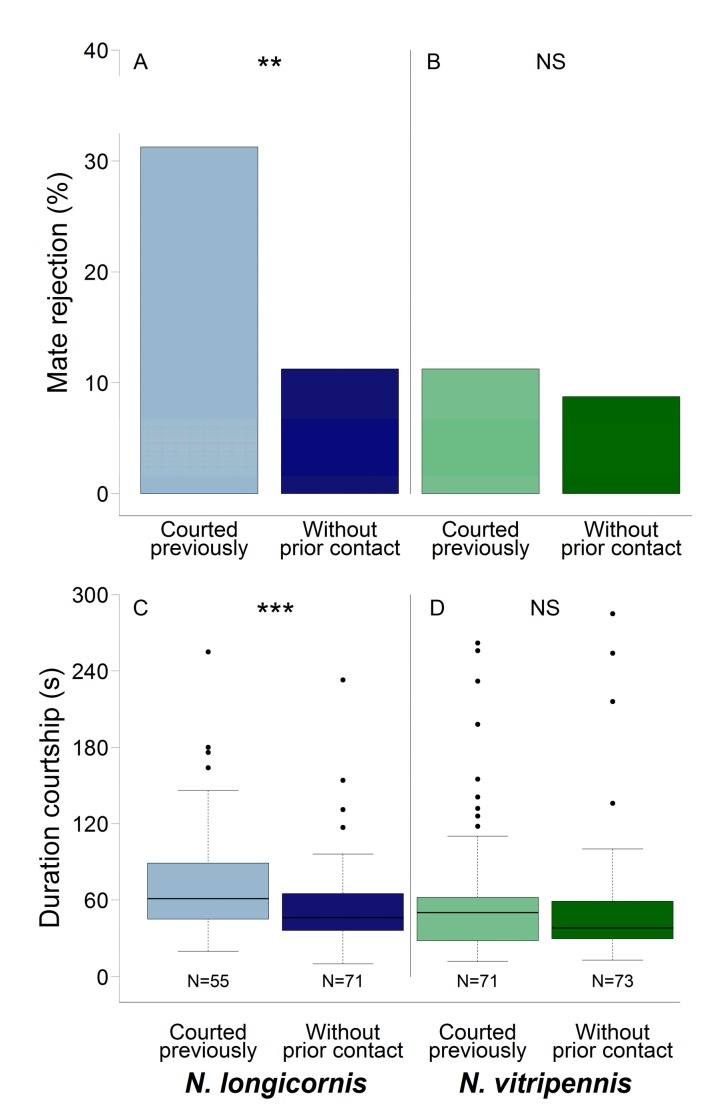
Females of *Nasonia longicornis* (**A**) rejected conspecific males more often and (**C**) consented to mating a conspecific male only after prolonged courtship when having been courted previously by a heterospecific male. In *N. vitripennis* females, heterospecific courtship had no effect on (**B**) female conspecific mate rejection, or (**D**) on the duration of courtship of the conspecific males. Boxplots display median (horizontal line within the box), lower and upper quartile (box margins), maximum/minimum range (whiskers; <1.5× above box height), and outliers (dots; ≥1.5× above box height). Asterisks indicate significant differences: Chi-square test (**A,B**) and Mann–Whitney U test (**C,D**): ** *p* < 0.01, *** *p* < 0.001, NS: Not significant.

*Nl* females that have been courted by *Nv* males rejected conspecific males more often than females without prior heterospecific contact (Chi-square test: χ_1_ = 9.56, *n* = 80 each, *p* < 0.01; Figure 1A; all raw data are provided in Table S1). In *Nv* females, heterospecific courtship had no effect on subsequent conspecific mate rejection (Chi-square test: χ_1_ = 0.28, *n* = 80 each, *p* = 0.60: Figure 1B).

### 3.2. Duration of Courtship

Previously courted *Nl* females that subsequently consented to mating a conspecific male did so after prolonged courtship compared to females without prior heterospecific contact (Mann–Whitney U test: U = 2206.5, *n* = 53 (*Nl* courted) and 61 (*Nl* without prior contact), *p* < 0.001; Figure 1C). In *Nv* females, heterospecific courtship had no effect on the subsequent duration of courtship by conspecific males (Mann–Whitney U test: U = 2283, *n* = 69 (*Nv* courted) and 63 (*Nv* without prior contact), *p* = 0.62; Figure 1D).

### 3.3. Aggression

Aggression by heterospecific males had no effect on *Nl* female mate rejection (Chi-square test: χ_1_ = 0.24, *N* = 32 (aggressive contact) and 48 (non-aggressive contact), *p* = 0.62) or the duration of subsequent conspecific courtship (Mann–Whitney U test: U = 812, *N* = 21 (aggressive) and 32 (non-aggressive), *p* = 0.23).

## 4. Discussion

*Nl* females were more reluctant to mate a conspecific after having been courted by a heterospecific male. This was reflected in both an increased conspecific mate rejection rate and an increased duration of courtship needed to induce receptivity in couples in which copulation took place. This reluctance to mate a conspecific was not a result of the aggressive attacks exhibited by *Nv* males towards *Nl* females. Hence, it is likely that *Nl* females use previous contact with and/or courtship by heterospecific males to assess the future risk of mismating. *Nl* females alerted by previous heterospecific courtship adjust their general mating behaviour and show an increased rejection of even conspecific males in subsequent courtship encounters. However, whether the mere contact with a heterospecific male, e.g., antennal contact, or the exposure to heterospecific courtship induces this adjustment remains to be investigated.

Rejecting conspecific males more frequently and consenting to conspecific copulation only after prolonged courtship likely results in *Nl* females losing valuable time that could otherwise be spent on the location of new host patches. In addition, it potentially increases the risk of leaving the natal host patch unmated. However, several males usually emerge from a host patch, and unmated females are potentially courted by several males prior to dispersal. In addition, at least in *Nv*, females become restless and switch to host-seeking behaviour only after mating [23,25]. Consistently, unmated *Nv* females have been rarely found to oviposit on new host patches in nature [24].

In contrast to *Nl* females, conspecific mate acceptance in *Nv* females was not affected by prior heterospecific courtship. A possible reason for this difference between the two species is that *Nv* females follow a different strategy to counteract the costs arising from interspecific mating. *Nv* females that have copulated with a heterospecific (*N. giraulti*, *Ng*) male show an increased willingness to remate with a conspecific. By remating, *Nv* females are able to produce female offspring numbers that are similar to those found in females that mated only once with a conspecific [25]. However, whether females of *Nl* exhibit similar remating adjustments needs further investigation. Furthermore, the costs of heterospecific copulation imposed on females of the two species likely differ in another aspect. While cytoplasmic incompatibility induced by the infection with strains of *Wolbachia* leads to a conversion to male offspring in *Nv*, it predominantly leads to mortality in *Nl* [36]. As a result, *Nl* females that have copulated with a heterospecific male most likely suffer higher fitness costs than *Nv* females that consented to interspecific copulation.

Mate discrimination is a plastic behavioural trait in *Nasonia*. Females adjust their mate acceptance rate according to their internal state and previous experience: they become less choosy with age (*Nv* and *Ng*) [25,28], refrain from mating when they have already mated with a conspecific (*Nv*) [37], show increased remating frequencies when having mated with a heterospecific male before (*Nv*) [25], and as shown here, are able to adjust mate acceptance in response to having experienced courtship by heterospecific males (*Nl*).

Plasticity in mate discrimination in response to being held in the presence of heterospecific mating partners has been demonstrated in various animal taxa. Females of the silverleaf whitefly *Bemisia tabaci* increase conspecific mate acceptance when males of a different biotype are present in the environment [16]. Females and males of three-spined sticklebacks (*Gasterosteus* spp.) become more discriminating against heterospecific partners when reared in mixed populations [17]. Males of the Trinidadian guppies *Poecilia reticulata* and *P. picta* as well as males of the fruit fly *Drosophila persimilis* learn to distinguish between conspecific and heterospecific females when held in mixed populations [40,41]. However, in contrast to *Nl*, individuals in these studies developed more accurate mate discrimination abilities in response to the presence of a reproductively interfering species. As females of *Nl* usually mate only once during their lifetime, a single mistake in mate choice is extremely costly for them, particularly if it is not, similar to in *Nv*, counteracted by increased remating after interspecific copulation. Instead of relying on the relatively slow process of learning by repeated experience, it may thus be more advantageous for *Nl* females to become more selective the moment that they recognize the presence of the interfering species. Recent studies indicate that the four *Nasonia* species differ in far more behavioural and ecological aspects than assumed so far, and that species interactions are far more complex than described to date [25,28,42,43,44,45,46]. In general, the potential role that the behavioural plasticity of mate discrimination can play in interactions and dynamics among co-occurring species has until now been widely neglected. The *Nasonia* model system offers fruitful opportunities for future investigations of plastic mate discrimination and species interactions on evolutionary, ecological, and behavioural levels, as well as on the level of underlying neurobiological and physiological processes.

## 5. Conclusions

After having been unsuccessfully courted by a heterospecific male, females of Nl are more reluctant to mate with a conspecific. In contrast, females of Nv do not change their mate acceptance behaviour following unsuccessful heterospecific courtship. This difference in the plasticity of mate acceptance between the two species is reasonable, because Nl females likely face higher fitness costs when consenting to interspecific copulation, and Nv females counteract the costs of mismating additionally through increased re-mating rates.

## Supplementary Materials

The following are available online at http://www.mdpi.com/2075-4450/9/3/112/s1, Table S1: Raw data.
