# Supplementary material for: Previous Interspecific Courtship Impairs Female Receptivity to Conspecifics in the Parasitoid Wasp Nasonia longicornis But Not in N. vitripennis
Source: Insects. 2018 Sep 1;9(3):112. doi: 10.3390/insects9030112 (PMC6163980; doi:10.3390/insects9030112)
Supplement: Supplementary file 1 [file insects-09-00112-s001.zip › Mair et al._raw.data_SOM.pdf]

Previous interspecific courtship impairs female receptivity to conspecifics in the parasitoid wasp *Nasonia longicornis* but not in *N. vitripennis*

Magdalena M. Mair, Nicole Seifert and Joachim Ruther
Institute of Zoology, University of Regensburg, Universitätsstraße 31, Regensburg D-93053, Germany

Author for correspondence: J. Ruther, e-mail: joachim.ruther @ur.de

FID	ID	treatment	female.sp	het.courtship	aggression	mated.consp	court.duration(s)
1	NvNv_01	Nv.without	Nv	no	no	yes	24
2	NlNl_01	Nl.without	Nl	no	no	yes	29
3	NvNl_01	Nv.courted	Nv	yes	no	yes	50
4	NlNv_01	Nl.courted	Nl	yes	yes	yes	164
5	NvNv_02	Nv.without	Nv	no	no	yes	30
6	NlNl_02	Nl.without	Nl	no	no	yes	35
7	NvNl_02	Nv.courted	Nv	yes	no	yes	232
8	NlNv_02	Nl.courted	Nl	yes	no	yes	35
9	NvNv_03	Nv.without	Nv	no	no	yes	13
10	NlNl_03	Nl.without	Nl	no	no	yes	154
11	NvNl_03	Nv.courted	Nv	yes	no	no	NA
12	NlNv_03	Nl.courted	Nl	yes	no	yes	97
13	NvNv_04	Nv.without	Nv	no	no	yes	21
14	NlNl_04	Nl.without	Nl	no	no	yes	30
15	NvNl_04	Nv.courted	Nv	yes	no	yes	22
16	NlNv_04	Nl.courted	Nl	yes	yes	yes	34
17	NvNv_05	Nv.without	Nv	no	no	yes	58
18	NlNl_05	Nl.without	Nl	no	no	yes	40
19	NvNl_05	Nv.courted	Nv	yes	no	yes	110
20	NlNv_05	Nl.courted	Nl	yes	yes	no	NA
21	NvNv_06	Nv.without	Nv	no	no	yes	41
22	NlNl_06	Nl.without	Nl	no	no	yes	10
23	NvNl_06	Nv.courted	Nv	yes	no	yes	16
24	NlNv_06	Nl.courted	Nl	yes	yes	yes	180
25	NvNv_07	Nv.without	Nv	no	no	yes	28
26	NlNl_07	Nl.without	Nl	no	no	yes	23
27	NvNl_07	Nv.courted	Nv	yes	no	no	NA
28	NlNv_07	Nl.courted	Nl	yes	no	yes	66
29	NvNv_08	Nv.without	Nv	no	no	yes	28
30	NlNl_08	Nl.without	Nl	no	no	yes	44
31	NvNl_08	Nv.courted	Nv	yes	no	yes	118
32	NlNv_08	Nl.courted	Nl	yes	no	yes	176
33	NvNv_09	Nv.without	Nv	no	no	yes	36
34	NlNl_09	Nl.without	Nl	no	no	yes	66
35	NvNl_09	Nv.courted	Nv	yes	no	no	NA
36	NlNv_09	Nl.courted	Nl	yes	yes	no	NA
37	NvNv_10	Nv.without	Nv	no	no	yes	57
38	NlNl_10	Nl.without	Nl	no	no	no	NA
39	NvNl_10	Nv.courted	Nv	yes	no	yes	65
40	NlNv_10	Nl.courted	Nl	yes	yes	yes	47
41	NvNv_11	Nv.without	Nv	no	no	yes	94
42	NlNl_11	Nl.without	Nl	no	no	yes	36
43	NvNl_11	Nv.courted	Nv	yes	no	yes	14
44	NlNv_11	Nl.courted	Nl	yes	yes	yes	45
45	NvNv_12	Nv.without	Nv	no	no	yes	32
46	NlNl_12	Nl.without	Nl	no	no	no	NA
47	NvNl_12	Nv.courted	Nv	yes	no	yes	86
48	NlNv_12	Nl.courted	Nl	yes	no	yes	37
49	NvNv_13	Nv.without	Nv	no	no	yes	29

50	NlNl_13	Nl.without	Nl	no	no	yes	80
51	NvNl_13	Nv.courted	Nv	yes	no	yes	21
52	NlNv_13	Nl.courted	Nl	yes	no	no	NA
53	NvNv_14	Nv.without	Nv	no	no	yes	24
54	NlNl_14	Nl.without	Nl	no	no	yes	233
55	NvNl_14	Nv.courted	Nv	yes	no	no	NA
56	NlNv_14	Nl.courted	Nl	yes	no	no	NA
57	NvNv_15	Nv.without	Nv	no	no	yes	29
58	NlNl_15	Nl.without	Nl	no	no	yes	40
59	NvNl_15	Nv.courted	Nv	yes	no	yes	60
60	NlNv_15	Nl.courted	Nl	yes	no	yes	53
61	NvNv_16	Nv.without	Nv	no	no	yes	136
62	NlNl_16	Nl.without	Nl	no	no	yes	40
63	NvNl_16	Nv.courted	Nv	yes	no	yes	35
64	NlNv_16	Nl.courted	Nl	yes	yes	yes	48
65	NvNv_17	Nv.without	Nv	no	no	yes	49
66	NlNl_17	Nl.without	Nl	no	no	yes	37
67	NvNl_17	Nv.courted	Nv	yes	no	yes	62
68	NlNv_17	Nl.courted	Nl	yes	no	yes	89
69	NvNv_18	Nv.without	Nv	no	no	yes	42
70	NlNl_18	Nl.without	Nl	no	no	yes	37
71	NvNl_18	Nv.courted	Nv	yes	no	yes	58
72	NlNv_18	Nl.courted	Nl	yes	no	yes	56
73	NvNv_19	Nv.without	Nv	no	no	yes	76
74	NlNl_19	Nl.without	Nl	no	no	yes	20
75	NvNl_19	Nv.courted	Nv	yes	no	yes	13
76	NlNv_19	Nl.courted	Nl	yes	yes	yes	61
77	NvNv_20	Nv.without	Nv	no	no	no	NA
78	NlNl_20	Nl.without	Nl	no	no	yes	56
79	NvNl_20	Nv.courted	Nv	yes	no	yes	54
80	NlNv_20	Nl.courted	Nl	yes	no	yes	70
81	NvNv_21	Nv.without	Nv	no	no	yes	285
82	NlNl_21	Nl.without	Nl	no	no	yes	46
83	NvNl_21	Nv.courted	Nv	yes	no	yes	60
84	NlNv_21	Nl.courted	Nl	yes	yes	yes	69
85	NvNv_22	Nv.without	Nv	no	no	yes	39
86	NlNl_22	Nl.without	Nl	no	no	yes	42
87	NvNl_22	Nv.courted	Nv	yes	no	yes	29
88	NlNv_22	Nl.courted	Nl	yes	yes	no	NA
89	NvNv_23	Nv.without	Nv	no	no	yes	38
90	NlNl_23	Nl.without	Nl	no	no	yes	36
91	NvNl_23	Nv.courted	Nv	yes	no	yes	15
92	NlNv_23	Nl.courted	Nl	yes	no	yes	51
93	NvNv_24	Nv.without	Nv	no	no	yes	64
94	NlNl_24	Nl.without	Nl	no	no	yes	34
95	NvNl_24	Nv.courted	Nv	yes	no	yes	256
96	NlNv_24	Nl.courted	Nl	yes	no	no	NA
97	NvNv_25	Nv.without	Nv	no	no	yes	41
98	NlNl_25	Nl.without	Nl	no	no	yes	21
99	NvNl_25	Nv.courted	Nv	yes	no	yes	262
100	NlNv_25	Nl.courted	Nl	yes	no	yes	73
101	NvNv_26	Nv.without	Nv	no	no	yes	36
102	NlNl_26	Nl.without	Nl	no	no	no	NA
103	NvNl_26	Nv.courted	Nv	yes	no	yes	60
104	NlNv_26	Nl.courted	Nl	yes	no	yes	90
105	NvNv_27	Nv.without	Nv	no	no	yes	15
106	NlNl_27	Nl.without	Nl	no	no	yes	51
107	NvNl_27	Nv.courted	Nv	yes	no	yes	30
108	NlNv_27	Nl.courted	Nl	yes	no	no	NA

109	NvNv_28	Nv.without	Nv	no	no	yes	38
110	NlNl_28	Nl.without	Nl	no	no	yes	117
111	NvNl_28	Nv.courted	Nv	yes	no	no	NA
112	NlNv_28	Nl.courted	Nl	yes	yes	yes	43
113	NvNv_29	Nv.without	Nv	no	no	no	NA
114	NlNl_29	Nl.without	Nl	no	no	no	47
115	NvNl_29	Nv.courted	Nv	yes	no	yes	NA
116	NlNv_29	Nl.courted	Nl	yes	no	no	NA
117	NvNv_30	Nv.without	Nv	no	no	yes	27
118	NlNl_30	Nl.without	Nl	no	no	yes	88
119	NvNl_30	Nv.courted	Nv	yes	no	yes	16
120	NlNv_30	Nl.courted	Nl	yes	yes	no	NA
121	NvNv_31	Nv.without	Nv	no	no	yes	76
122	NlNl_31	Nl.without	Nl	no	no	no	NA
123	NvNl_31	Nv.courted	Nv	yes	no	yes	55
124	NlNv_31	Nl.courted	Nl	yes	no	yes	51
125	NvNv_32	Nv.without	Nv	no	no	no	NA
126	NlNl_32	Nl.without	Nl	no	no	yes	69
127	NvNl_32	Nv.courted	Nv	yes	no	yes	70
128	NlNv_32	Nl.courted	Nl	yes	yes	yes	114
129	NvNv_33	Nv.without	Nv	no	no	yes	60
130	NlNl_33	Nl.without	Nl	no	no	no	NA
131	NvNl_33	Nv.courted	Nv	yes	no	yes	28
132	NlNv_33	Nl.courted	Nl	yes	no	no	NA
133	NvNv_34	Nv.without	Nv	no	no	yes	86
134	NlNl_34	Nl.without	Nl	no	no	yes	46
135	NvNl_34	Nv.courted	Nv	yes	no	yes	50
136	NlNv_34	Nl.courted	Nl	yes	yes	yes	51
137	NvNv_35	Nv.without	Nv	no	no	yes	216
138	NlNl_35	Nl.without	Nl	no	no	yes	20
139	NvNl_35	Nv.courted	Nv	yes	no	yes	23
140	NlNv_35	Nl.courted	Nl	yes	yes	yes	75
141	NvNv_36	Nv.without	Nv	no	no	yes	44
142	NlNl_36	Nl.without	Nl	no	no	yes	49
143	NvNl_36	Nv.courted	Nv	yes	no	yes	27
144	NlNv_36	Nl.courted	Nl	yes	yes	yes	102
145	NvNv_37	Nv.without	Nv	no	no	yes	35
146	NlNl_37	Nl.without	Nl	no	no	yes	32
147	NvNl_37	Nv.courted	Nv	yes	no	yes	59
148	NlNv_37	Nl.courted	Nl	yes	no	yes	67
149	NvNv_38	Nv.without	Nv	no	no	yes	21
150	NlNl_38	Nl.without	Nl	no	no	yes	36
151	NvNl_38	Nv.courted	Nv	yes	no	yes	19
152	NlNv_38	Nl.courted	Nl	yes	no	yes	32
153	NvNv_39	Nv.without	Nv	no	no	yes	35
154	NlNl_39	Nl.without	Nl	no	no	yes	61
155	NvNl_39	Nv.courted	Nv	yes	no	yes	29
156	NlNv_39	Nl.courted	Nl	yes	no	yes	31
157	NvNv_40	Nv.without	Nv	no	no	yes	100
158	NlNl_40	Nl.without	Nl	no	no	yes	51
159	NvNl_40	Nv.courted	Nv	yes	no	yes	51
160	NlNv_40	Nl.courted	Nl	yes	yes	yes	82
161	NvNv_41	Nv.without	Nv	no	no	yes	34
162	NlNl_41	Nl.without	Nl	no	no	yes	30
163	NvNl_41	Nv.courted	Nv	yes	no	yes	60
164	NlNv_41	Nl.courted	Nl	yes	yes	no	NA
165	NvNv_42	Nv.without	Nv	no	no	yes	34
166	NlNl_42	Nl.without	Nl	no	no	yes	43
167	NvNl_42	Nv.courted	Nv	yes	no	no	NA

168	NlNv_42	Nl.courted	Nl	yes	yes	no	NA
169	NvNv_43	Nv.without	Nv	no	no	yes	30
170	NlNl_43	Nl.without	Nl	no	no	yes	56
171	NvNl_43	Nv.courted	Nv	yes	no	yes	19
172	NlNv_43	Nl.courted	Nl	yes	no	yes	76
173	NvNv_44	Nv.without	Nv	no	no	yes	26
174	NlNl_44	Nl.without	Nl	no	no	yes	50
175	NvNl_44	Nv.courted	Nv	yes	no	yes	37
176	NlNv_44	Nl.courted	Nl	yes	yes	yes	42
177	NvNv_45	Nv.without	Nv	no	no	yes	25
178	NlNl_45	Nl.without	Nl	no	no	yes	52
179	NvNl_45	Nv.courted	Nv	yes	no	yes	31
180	NlNv_45	Nl.courted	Nl	yes	no	yes	146
181	NvNv_46	Nv.without	Nv	no	no	yes	56
182	NlNl_46	Nl.without	Nl	no	no	yes	44
183	NvNl_46	Nv.courted	Nv	yes	no	yes	56
184	NlNv_46	Nl.courted	Nl	yes	yes	yes	53
185	NvNv_47	Nv.without	Nv	no	no	yes	48
186	NlNl_47	Nl.without	Nl	no	no	yes	28
187	NvNl_47	Nv.courted	Nv	yes	no	yes	132
188	NlNv_47	Nl.courted	Nl	yes	yes	yes	133
189	NvNv_48	Nv.without	Nv	no	no	yes	52
190	NlNl_48	Nl.without	Nl	no	no	yes	45
191	NvNl_48	Nv.courted	Nv	yes	no	yes	25
192	NlNv_48	Nl.courted	Nl	yes	no	no	NA
193	NvNv_49	Nv.without	Nv	no	no	yes	24
194	NlNl_49	Nl.without	Nl	no	no	yes	48
195	NvNl_49	Nv.courted	Nv	yes	no	yes	57
196	NlNv_49	Nl.courted	Nl	yes	yes	yes	60
197	NvNv_50	Nv.without	Nv	no	no	yes	37
198	NlNl_50	Nl.without	Nl	no	no	yes	16
199	NvNl_50	Nv.courted	Nv	yes	no	no	NA
200	NlNv_50	Nl.courted	Nl	yes	yes	no	NA
201	NvNv_51	Nv.without	Nv	no	no	yes	53
202	NlNl_51	Nl.without	Nl	no	no	yes	40
203	NvNl_51	Nv.courted	Nv	yes	no	yes	60
204	NlNv_51	Nl.courted	Nl	yes	no	yes	50
205	NvNv_52	Nv.without	Nv	no	no	yes	62
206	NlNl_52	Nl.without	Nl	no	no	yes	27
207	NvNl_52	Nv.courted	Nv	yes	no	no	NA
208	NlNv_52	Nl.courted	Nl	yes	no	yes	39
209	NvNv_53	Nv.without	Nv	no	no	yes	33
210	NlNl_53	Nl.without	Nl	no	no	yes	48
211	NvNl_53	Nv.courted	Nv	yes	no	yes	45
212	NlNv_53	Nl.courted	Nl	yes	yes	no	NA
213	NvNv_54	Nv.without	Nv	no	yes	no	NA
214	NlNl_54	Nl.without	Nl	no	no	no	NA
215	NvNl_54	Nv.courted	Nv	yes	no	yes	155
216	NlNv_54	Nl.courted	Nl	yes	no	yes	255
217	NvNv_55	Nv.without	Nv	no	no	yes	28
218	NlNl_55	Nl.without	Nl	no	no	no	NA
219	NvNl_55	Nv.courted	Nv	yes	no	yes	12
220	NlNv_55	Nl.courted	Nl	yes	no	no	NA
221	NvNv_56	Nv.without	Nv	no	no	yes	32
222	NlNl_56	Nl.without	Nl	no	no	no	NA
223	NvNl_56	Nv.courted	Nv	yes	no	yes	83
224	NlNv_56	Nl.courted	Nl	yes	yes	no	NA
225	NvNv_57	Nv.without	Nv	no	no	yes	254
226	NlNl_57	Nl.without	Nl	no	no	yes	39

227	NvNl_57	Nv.courted	Nv	yes	no	yes	30
228	NlNv_57	Nl.courted	Nl	yes	yes	no	NA
229	NvNv_58	Nv.without	Nv	no	no	yes	62
230	NlNl_58	Nl.without	Nl	no	no	yes	86
231	NvNl_58	Nv.courted	Nv	yes	no	yes	19
232	NlNv_58	Nl.courted	Nl	yes	no	no	NA
233	NvNv_59	Nv.without	Nv	no	no	no	NA
234	NlNl_59	Nl.without	Nl	no	no	yes	89
235	NvNl_59	Nv.courted	Nv	yes	no	no	NA
236	NlNv_59	Nl.courted	Nl	yes	yes	yes	20
237	NvNv_60	Nv.without	Nv	no	no	no	NA
238	NlNl_60	Nl.without	Nl	no	no	yes	47
239	NvNl_60	Nv.courted	Nv	yes	no	yes	39
240	NlNv_60	Nl.courted	Nl	yes	no	yes	67
241	NvNv_61	Nv.without	Nv	no	no	yes	21
242	NlNl_61	Nl.without	Nl	no	no	yes	72
243	NvNl_61	Nv.courted	Nv	yes	no	yes	28
244	NlNv_61	Nl.courted	Nl	yes	no	yes	129
245	NvNv_62	Nv.without	Nv	no	no	yes	41
246	NlNl_62	Nl.without	Nl	no	no	yes	82
247	NvNl_62	Nv.courted	Nv	yes	no	yes	62
248	NlNv_62	Nl.courted	Nl	yes	no	no	NA
249	NvNv_63	Nv.without	Nv	no	no	yes	66
250	NlNl_63	Nl.without	Nl	no	no	yes	131
251	NvNl_63	Nv.courted	Nv	yes	no	yes	31
252	NlNv_63	Nl.courted	Nl	yes	no	yes	164
253	NvNv_64	Nv.without	Nv	no	no	yes	83
254	NlNl_64	Nl.without	Nl	no	no	yes	72
255	NvNl_64	Nv.courted	Nv	yes	no	yes	48
256	NlNv_64	Nl.courted	Nl	yes	no	no	NA
257	NvNv_65	Nv.without	Nv	no	no	yes	46
258	NlNl_65	Nl.without	Nl	no	no	yes	65
259	NvNl_65	Nv.courted	Nv	yes	no	yes	126
260	NlNv_65	Nl.courted	Nl	yes	no	yes	45
261	NvNv_66	Nv.without	Nv	no	no	no	NA
262	NlNl_66	Nl.without	Nl	no	no	yes	NA
263	NvNl_66	Nv.courted	Nv	yes	no	yes	18
264	NlNv_66	Nl.courted	Nl	yes	no	yes	128
265	NvNv_67	Nv.without	Nv	no	no	yes	NA
266	NlNl_67	Nl.without	Nl	no	no	yes	NA
267	NvNl_67	Nv.courted	Nv	yes	no	yes	68
268	NlNv_67	Nl.courted	Nl	yes	no	yes	43
269	NvNv_68	Nv.without	Nv	no	no	yes	NA
270	NlNl_68	Nl.without	Nl	no	no	yes	NA
271	NvNl_68	Nv.courted	Nv	yes	no	yes	75
272	NlNv_68	Nl.courted	Nl	yes	yes	yes	52
273	NvNv_69	Nv.without	Nv	no	no	yes	NA
274	NlNl_69	Nl.without	Nl	no	no	yes	NA
275	NvNl_69	Nv.courted	Nv	yes	no	yes	141
276	NlNv_69	Nl.courted	Nl	yes	no	yes	88
277	NvNv_70	Nv.without	Nv	no	no	yes	NA
278	NlNl_70	Nl.without	Nl	no	no	yes	NA
279	NvNl_70	Nv.courted	Nv	yes	no	yes	38
280	NlNv_70	Nl.courted	Nl	yes	yes	yes	38
281	NvNv_71	Nv.without	Nv	no	no	yes	NA
282	NlNl_71	Nl.without	Nl	no	no	yes	NA
283	NvNl_71	Nv.courted	Nv	yes	no	yes	50
284	NlNv_71	Nl.courted	Nl	yes	no	no	NA
285	NvNv_72	Nv.without	Nv	no	no	yes	NA

286	NlNl_72	Nl.without	Nl	no	no	yes	NA
287	NvNl_72	Nv.courted	Nv	yes	no	yes	26
288	NlNv_72	Nl.courted	Nl	yes	no	yes	45
289	NvNv_73	Nv.without	Nv	no	no	yes	NA
290	NlNl_73	Nl.without	Nl	no	no	yes	NA
291	NvNl_73	Nv.courted	Nv	yes	no	yes	79
292	NlNv_73	Nl.courted	Nl	yes	no	yes	69
293	NvNv_74	Nv.without	Nv	no	no	yes	35
294	NlNl_74	Nl.without	Nl	no	no	yes	NA
295	NvNl_74	Nv.courted	Nv	yes	no	yes	198
296	NlNv_74	Nl.courted	Nl	yes	no	yes	65
297	NvNv_75	Nv.without	Nv	no	no	yes	30
298	NlNl_75	Nl.without	Nl	no	no	yes	NA
299	NvNl_75	Nv.courted	Nv	yes	no	yes	30
300	NlNv_75	Nl.courted	Nl	yes	no	yes	60
301	NvNv_76	Nv.without	Nv	no	no	yes	76
302	NlNl_76	Nl.without	Nl	no	no	yes	80
303	NvNl_76	Nv.courted	Nv	yes	no	yes	48
304	NlNv_76	Nl.courted	Nl	yes	no	no	NA
305	NvNv_77	Nv.without	Nv	no	no	yes	50
306	NlNl_77	Nl.without	Nl	no	no	yes	48
307	NvNl_77	Nv.courted	Nv	yes	no	yes	30
308	NlNv_77	Nl.courted	Nl	yes	no	no	NA
309	NvNv_78	Nv.without	Nv	no	no	yes	NA
310	NlNl_78	Nl.without	Nl	no	no	yes	96
311	NvNl_78	Nv.courted	Nv	yes	no	yes	38
312	NlNv_78	Nl.courted	Nl	yes	yes	no	NA
313	NvNv_79	Nv.without	Nv	no	no	yes	NA
314	NlNl_79	Nl.without	Nl	no	no	yes	41
315	NvNl_79	Nv.courted	Nv	yes	no	yes	NA
316	NlNv_79	Nl.courted	Nl	yes	no	yes	NA
317	NvNv_80	Nv.without	Nv	no	no	yes	NA
318	NlNl_80	Nl.without	Nl	no	no	yes	NA
319	NvNl_80	Nv.courted	Nv	yes	no	yes	61
320	NlNv_80	Nl.courted	Nl	yes	no	yes	NA
